# Surface characteristics and molecular interactions of thin films between bubbles by molecular simulations

**DOI:** 10.3389/fchem.2024.1493571

**Published:** 2025-01-06

**Authors:** Tiefeng Peng, Yangyang Huai

**Affiliations:** ^1^ Jiangxi Copper Technology Institute Co., Ltd, Nanchang, Jiangxi, China; ^2^ Jiangxi Copper Corporation, Nanchang, Jiangxi, China

**Keywords:** molecular modeling, ultra-stable froth, froth flotation, bubble coalescence, froth stability

## Abstract

**Introduction:**

Whether in industrial production or daily life, froth plays an important role in many processes. Sometimes, froth exists as a necessity and is also regarded as the typical characteristic of products, e.g., froth on shampoo. Froth often makes an important contribution to product performance, such as in cleaning operations. On the other hand, froth may destroy the production process, such as in the textile and paper industry. Another example, ultra-stable froth accumulates on the thickener from flotation brings a series of difficulties to pumping, settling and dewatering operations, and would lead to pollution to the industrial circulating water treatment, thus it must be prevented.

**Methods:**

In this work, the factors affecting the stability of froth, and relationship of bubble coalescence and film rupture was investigated, and molecular simulations (MD) were performed to study the aqueous molecular formation and surface characteristics of thin films between bubbles that contribute to the froth stability.

**Results:**

The detailed interfacial structure, molecular formation along *Z*-axis, angle distribution within the first and second layer, and also critical thickness were studied and discussed. The film rupture was validated and interpreted by the water-water interactions within the thin film, and these surface interactions were also examined using binding energy, dipole autocorrelation function (DAF). These simulations explicitly utilize polarizable potential model, incorporating many-body interactions, in which induced polarization plays a critical role in reproducing experimental observables and understanding physical behavior.

**Discussion:**

The results provide beneficial insight for ultra-stable removal from microscopic view, and have direct benefits in dissolved air flotation used in mining industry, to develop efficient and sustainable processes for industries to minimize water and chemical usage.

## 1 Introduction

Foam can be defined as gas dispersed in a liquid, and gas accounts for most of the volume in foam. Foam is thermodynamically unstable and will gradually decay. The time range of attenuation varies greatly. The short-lived foam decays and then stabilizes in a few seconds through the surfactant. Foam stabilized by polymers or surfactants may last for hours or even days. Most of the foam encountered in practice involves aqueous solutions.

Compared with their macroscopic counterparts, nano-bubbles and thin films (Cazabat et al., 1991; Cong and Cao, 2004; Ewing, 2004; Ida and Miksis, 1996) exhibit many different properties and play an important role in the fields of surface chemistry and chemical industry. For example, interfacial nano-bubbles can reduce the flow resistance of nano-fluids, increase the recovery of mineral flotation (Jing Yu and Song, 2021; Minghui, 2022) (Cheng Guozhu and Zhang, 2022), and remove surface contaminants. A pure liquid generally would not produce bubbles alone, as the film cannot be stable without an adsorption layer on the surface of the liquid. However, surfactants, which have stable foaming properties, could be added.

Depending on the type and concentration of stabilizers, the adsorption layer may be either flowing or non-flowing. The non-flowing adsorption layer is usually generated in the polymer, which tends to make the foam more stable. Foam stability (QiCheng and Jin, 2006; Rusanov et al., 1998; Wang et al., 2015; Zhou et al., 2014) refers to the maintenance time of foam from generation to extinction and the self-repair ability after external stimulation. The main influencing factors include surface tension, surface viscosity, liquid viscosity, Marangoni effect, and surface expansion elastic modulus. The stability and decay of foam are generally determined by a variety of mechanisms (Bai et al., 2018; Fan et al., 2013; Hédreul and Frens, 2001; Jong and Quoc Phuc, 2018). Some are used to isolate thin films, and the connection points between thin films also play an important role.

An ultra-stable foam (Chen et al., 2023; Du et al., 2017; Lv et al., 2014; Sheng et al., 2020) from flotation, with strong stability, is difficult to break due to long-term accumulation in the thickener. Such foams exist widely in the mineral processing of mines, resulting in the loss of much non-ferrous metal and rare earth resources and causing mine wastewater pollution. To understand and interpret the stability and rupture mechanisms of froth, it is beneficial to study the microscopic structure and the corresponding molecular distribution within the film between bubbles (Dhiman and Chandra, 2008; Evers et al., 1996; Hwang et al., 1997; Liao et al., 2022; Shikhmurzaev, 2005; Zhang et al., 2023). This work adopts the molecular simulation method to investigate this aspect.

Molecular simulation usually applies Monte Carlo (MC) (Hammersley et al., 1965) and/or molecular dynamics (MD) simulations (Nosé, 1984; van Gunsteren and Berendsen, 1990). Molecular simulation is a method of simulating the structure and behavior of molecules by using computer models at the atomic level and then simulating the various physical and chemical properties of the molecular system. It is based on experiment and builds a set of models through basic principles (Grossfield et al., 2003; Ponder et al., 2010) and algorithms so as to calculate the reasonable molecular structure and molecular behavior. Molecular simulation can both simulate the static structure of molecules and simulate the dynamic behavior of molecular systems.

In this study, foam structure and factors influencing the stability of foam are analyzed. Modeling of nano-bubbles and nanofilms is presented, and the molecular interactions within the film are investigated using qualities such as binding energy and DAF. It is beneficial to investigate the microscopic aspects that contribute to bubble coalescence and film stability, and the systematic investigation provided molecular-level insights for the micro-nano scale film between liquid bubbles.

## 2 Modeling of thin films between bubbles

A methodology was proposed so that the bubble–bubble interaction (in Supplementary Figures S1, S2) could be modeled as the surface interaction of liquid films between bubbles that account. In turn, the gas–liquid interface was also an important aspect that should be noticed.

**FIGURE 1:**
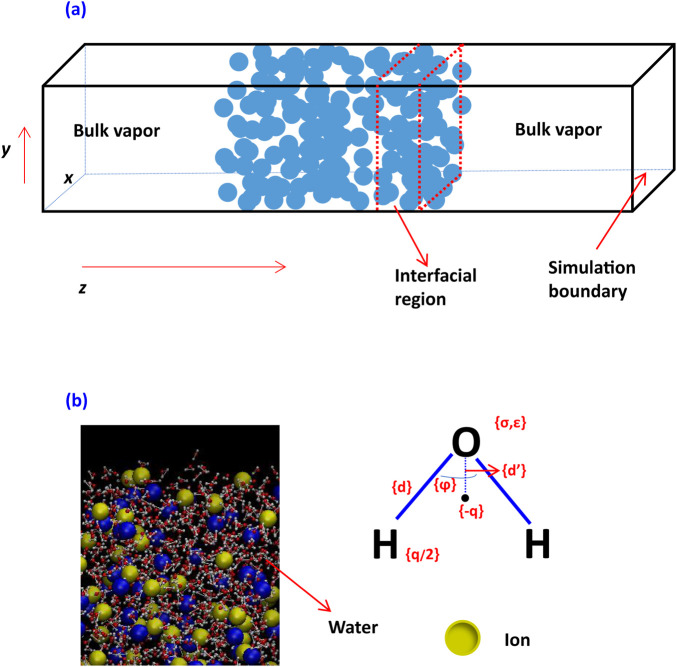
Modeling of an aqueous system with vapor, water film, and interfacial region **(A)** and ions in water **(B)**.

Molecular dynamics (MD) simulations were adopted in this study to explore the effects of dissolved ionic species on the interfacial region structure (as shown in Figure 1A) and critical thickness of thin liquid films. In these simulations, appropriate potential functions for each species will be used to model force interactions among water molecules and ions.

The detailed modeling of an aqueous system using MD is seen in Figure 1B, using explicit simulation of water where H has no Lennard–Jones (LJ) interaction. All atoms have Coulombic interaction. There is no Coulombic interaction for an LJ fluid.

Using classical MD simulation with a polarizable potential model (Chang et al., 1997; Dang and Chang, 1997), the equations for these interactions (Dang and Chang, 2003) are shown as Equations 1–3.

$$U_{\text{tot}}=U_{\text{pair}}+U_{\text{pol}}+U_{\mathrm{int}},$$ (1)

$$U_{\text{pair}}=\sum_{i}\sum_{j}\left(4\varepsilon \left[{\left(\frac{\sigma_{ij}}{r_{ij}}\right)}^{12}-{\left(\frac{\sigma_{ij}}{r_{ij}}\right)}^{6}\right]+\frac{q_{i}q_{j}}{r_{ij}}\right),$$ (2)

$$U_{\text{pol}}=U_{\text{stat}}+U_{\text{pp}}+U_{\text{self}}=-\sum_{i}\mu_{i}\cdot E_{i}^{0}-\frac{1}{2}\sum_{j}\sum_{j\neq 1}\mu_{i}\cdot T_{ij}\cdot \mu_{j}+\sum_{i}\frac{\mu_{i}\cdot \mu_{i}}{{2\alpha }_{i}}.$$ (3)

Here, σ and ε are the Lennard–Jones parameters, *r*_*ij*_ is the distance between site *i* and *j*, and *q* is the charge. *E*_*i*_^*0*^ is the electric field at site *i* produced by the fixed charges within the system, *µ*_*i*_ is the induced dipole moment at atom site *I*, and *T*_*ij*_ is the dipole tensor. The first term in Equation 3 is the charge–dipole interaction, the second term describes the dipole–dipole interaction, and the last term is the energy associated with the generation of the dipole moment *µ*_*i*_.

*U*_int_, bonded interactions, is intra-molecular potential, including bond *E*_bond_, angle *E*_angle_, and torsion interactions *E*_torsion_. The equations for these intra-molecular interactions are shown as Equations 4–6.

$$E_{bond}=\sum_{bonds}K_{r}{\left(r-r_{eq}\right)}^{2},$$ (4)

$$E_{angle}={\sum_{angles}K_{\theta }\left(\theta -\theta_{eq}\right)}^{2},$$ (5)

$$E_{torsion}=\sum_{i}\frac{V_{1}}{2}\left[1+\cos\left(\phi_{i}\right)\right]+\frac{V_{2}}{2}\left[1-\cos\left(2\phi_{i}\right)\right]+\frac{V_{3}}{2}\left[1+\cos\left(3\phi_{i}\right)\right].$$ (6)

Here, *r*_*eq*_ and *θ*_*eq*_ are equilibrium values of the bond length and bond angle. The geometric combining rules for the LJ coefficients (Peng et al., 2012) were employed as shown in Equation 7:

εij=εiiεjj12;σij=σiiσjj12. (7)

The coefficient *f*_*ij*_ = 0.0 for any *i*-*j* pairs connected by a valence bond (1–2 pairs) or a valence bond angle (1–3 pairs); *f*_*ij*_ = 0.5 for 1–4 pair interactions (atoms separated by exactly three bonds), and *f*_*ij*_ = 1.0 for all of the other cases.

## 3 Results and discussion

### 3.1 Surface characteristics of the liquid thin film and molecular formation

Classical DLVO theory did not consider the factors that arise from the microstructure of water molecules (Paunov et al., 1996) and regarded water as a uniform medium. As such, classical DLVO theory could not accurately identify the role of the short-range and strong repulsive interaction. In this study, the detailed parameters for water and ions employed are provided in Supplementary Table S1 for the interfacial structure, which can give reasonable structure and thermodynamics of the bulk and the air/liquid interface of water.

The MD simulations were performed on a system of water and cation halides (e.g., Na^+^, Cl^−^, I^−^). Ewald summation techniques were used. The LJ cutoff was 11 Å, and the time step was 2 fs. During the simulation, the SHAKE algorithm was applied to fix the water OH and HH bond lengths. Considering that periodic boundary conditions had a certain effect on the system, a 50 Å vacuum layer was added to the model along the *Z*-axis to reduce this effect. Molecular dynamics simulations (potential parameters in Supplementary Table S1) using a polarizable potential model for the aqueous system were applied, where r_m_ = σ * 2^1/6^. σ and ε are the Lennard–Jones parameters, *q* is the atomic charge, and *α* is the atomic polarizability. Molecular mechanics that explicitly account for induced polarization represent the next generation of physical models for molecular dynamics simulations.

**FIGURE 2:**
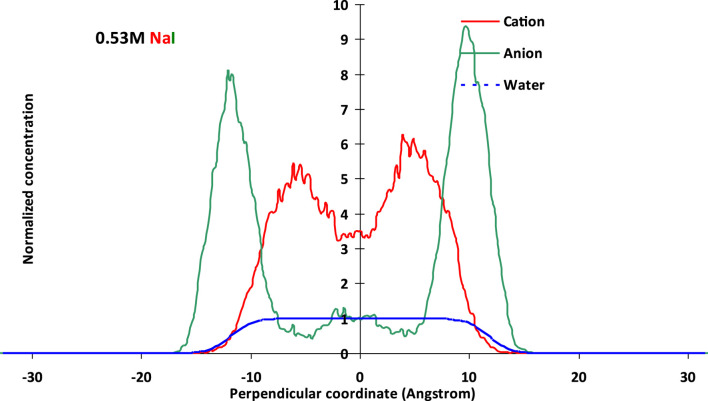
Normalized molecular distributions of cations and anions within thin films using a polarizable model with potential parameters listed in Supplementary Table S1 at 0.53 M NaCl (above) and 0.53 M NaI (below).

It is suggested that the molecular type and its partition of these molecules within the thin films have a great influence on the surface interaction, which in turn affects the final surface force (net force) of the liquid film and the foam stability that equilibrate the thin film (as in Supplementary Figure S2). The molecular basis of thin liquid films, in terms of molecule distribution of ions, for example, cation and anion, and water, are shown in Figure 2.

It could be observed from Figure 2 that the I^−^ anion showed higher normalized concentration strength than that of Cl^−^ at the same solution concentration and film thickness. By comparing water molecules within the films from the two types of halides, it could be found that NaCI tended to gather more along the bulk than NaI, on the whole. Cl^−^ or I^−^ both showed preference toward the vapor–liquid interface, though I^−^ exhibited more pronounced along the aqueous surface. This may be due to the larger polarizability from the polarizable potential, which could capture the surface sensitivity by simulation. The anion exhibited a slight adsorption by the film interface, and this anion preference would result in a change of the cation distribution, which would be more pronounced in thinner films.

**FIGURE 3:**
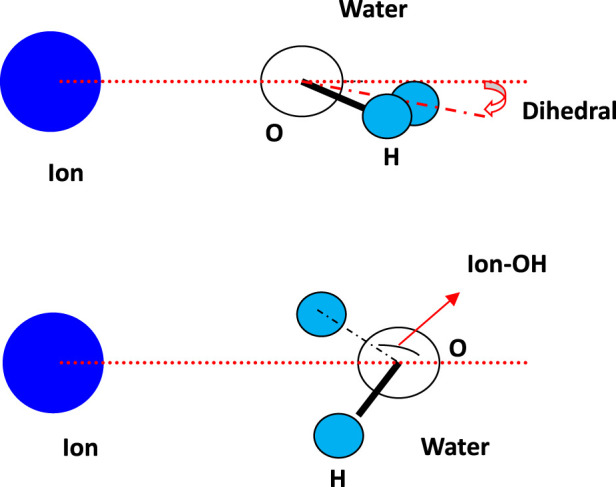
Schematic diagram of the angle between ion and water, denoted as dihedral, and the angle between ion-1/2OH, denoted as Ion-OH.

**FIGURE 4:**
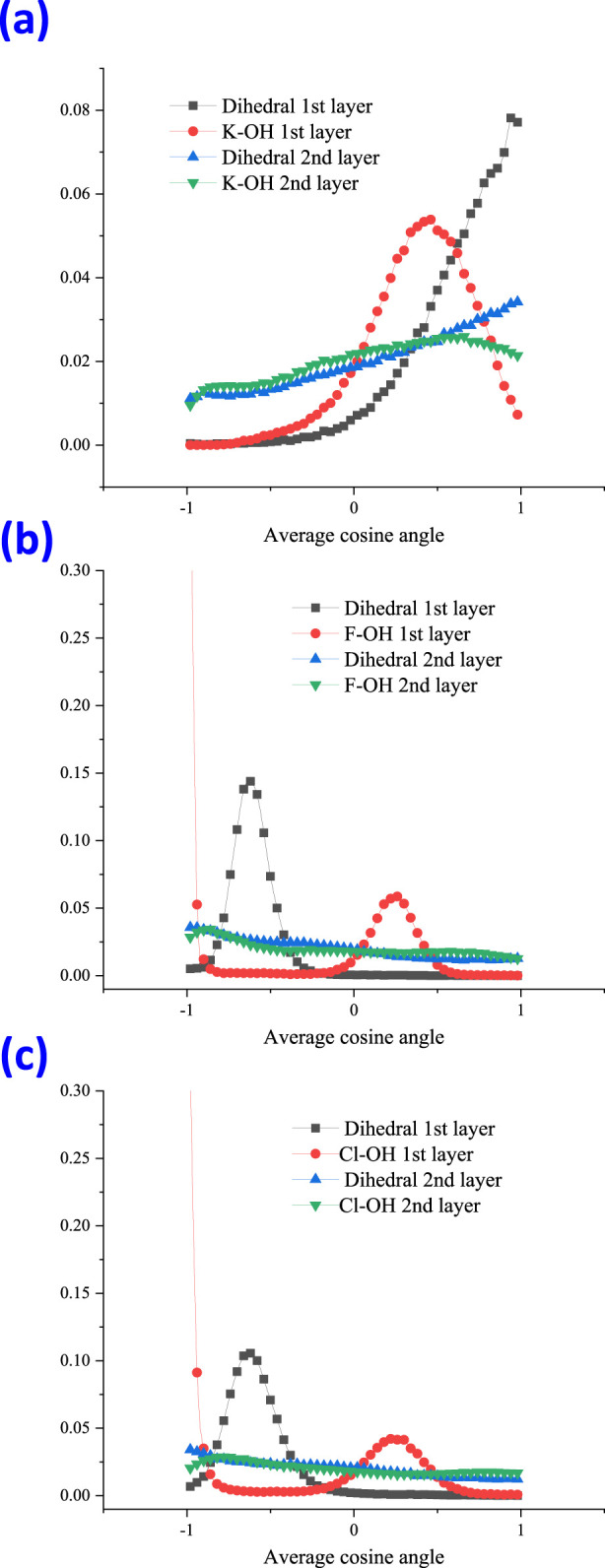
Angle distribution for cation K^+^ **(A)**, anion F^−^ **(B)**, and Cl^−^ **(C)**, at the 1st and 2nd layer with the dihedral angle and the angle of Ion-OH, shown as the value of the cosine angle.

In order to investigate the molecular orientation when running within the aqueous thin film, the angles between water molecules and ions were studied, and their schematic diagram related to water is shown in Figure 3. The angle between ion and water (above) is denoted as the dihedral angle, and the angle between ion-1/2OH is denoted as Ion-OH. The calculated angle distribution for cation K^+^ and the halide ions is shown in Figure 4. Within the 1st and 2nd layers, the obtained distribution is presented using the value of the cosine of the angle.

**FIGURE 5:**
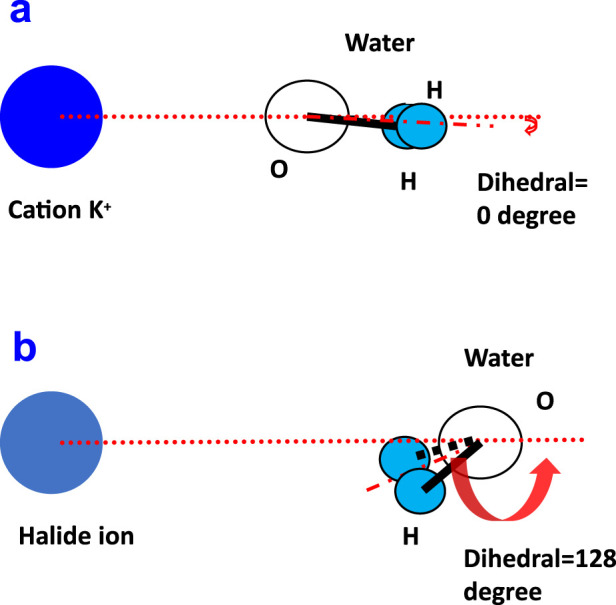
Maximum probability of the molecular orientation for the ion–water dihedral angle for cation K^+^ **(A)** and for a halide ion **(B)**.

From Figure 4, the angle distribution of the dihedral at the 1st (black line) for cation K^+^ was observed to be completely different from those of the halide ions. The dihedral angle at the 1st (black line) for halide ions F^−^ and Cl^−^ both showed a peak at a cosine value of around −0.6 (Figure 5B), which points to approximately 128°. However, for cation K^+^, the orientation of maximum probability nearly changed to 1, which corresponds to a dihedral angle of 0° (Figure 5A).

At the 2nd layer for the dihedral angle, the orientation of maximum probability was still toward 0° for cation K^+^. However, halide ions are nearly distributed randomly and showed no apparent preference. For the angle distribution of K^+^-OH, within the 1st layer, the orientation of the most probability lies at the peak of the cosine value 0.46, equal to an angle of 63°. For halide ions, the maximum probability of the angle of ion-OH lies at the peak of cosine value 0.24, corresponding to 76°. When extended to the 2nd layer, the ion-OH showed nearly random distribution for halide ions.

A radial distribution function (RDF) is a measure of the spatial distribution of particles in a system. An RDF is calculated by dividing the number of particles within a certain distance from a reference particle by the expected number of particles in that distance interval. This calculation is repeated for various distance intervals to obtain a complete RDF curve. RDF analysis is a method for studying the interaction between particles. By analyzing the RDF curve, the distribution pattern of particles at different distances can be understood. In the study of the hydration process, RDF could help to reveal the hydrogen bonding network structure and particle spacing distribution between water molecules. RDF or pair correlation function between particles of type *A* and *B* is defined according to Equation 8:

$$\begin{array}{l} g_{AB}\left(r\right)=\frac{\langle \rho_{B}\left(r\right)\rangle }{{\langle \rho_{B}\rangle }_{local}}\\ =\frac{1}{{\langle \rho_{B}\rangle }_{local}}\frac{1}{N_{A}}\sum_{i\in A}^{N_{A}}\sum_{j\in B}^{N_{B}}\frac{\left(r_{ij}-r\right)}{4\pi r^{2}}, \end{array}$$ (8)

where $\langle \rho_{B}\left(r\right)\rangle$ is the particle density of type *B* at a distance *r* around particles *A*, and $\left\langle \rho_{B}\rangle_{local}\right.$ is the particle density of type *B* averaged over all spheres around particle *A* with radius *r*_max_.

**FIGURE 6:**
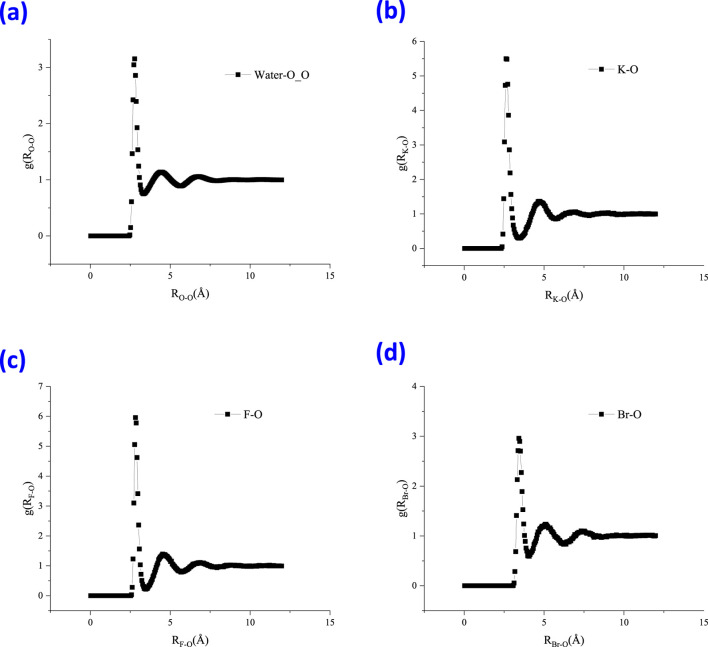
Radial distribution function (RDF) of water molecules with different species within the thin liquid film, **(A)** water-O_O, **(B)** K-O, **(C)** F-O, and **(D)** Br-O.

In this study, RDFs of water molecules with different species, such as K-O and the halide-O pairs, were examined and are shown in Figure 8. A first peak can be observed in Figure 6A at approximately 2.78 Å for the water O-O pair. The peak in the O-O radial distribution function occurs at roughly 2.8 Å, which is the well-known average hydrogen bond length in water. This also validated the good agreement of the molecular model with the experimental measurement. For the K-O pair, the first peak lies at approximately 2.63 Å. The first peak of the F-O pair lies at 2.83 Å with an intensity of 5.96; however, for the Br-O pair, it lies at 3.43 Å with an intensity of 2.96. For halide ions, the smaller the ion, the ‘tighter’ the ion-water structure will be, comparing Figures 6C,D. The coordination number can be regarded as the average number of water molecules within the range of the first shell, which could be obtained by the integral of g_IO_(R) within the first salvation shell distance.

Diffusion coefficients of ions are usually calculated experimentally from their conductivities. In simulations, two methods can be applied to calculate them. One method is the direct calculation of the mean square displacement of ions for a unit time period. Calculation of the velocity autocorrelation function also provides a link to the diffusion coefficient. However, given a long simulation period, the two methods give equivalent results for the diffusion coefficients. In a molecular dynamics calculation, the self-diffusion coefficient can, in principle, be calculated by monitoring the mean square displacement of the ion and exploiting the well-known relation, the Einstein relation, to calculate mean-squared displacement (MSD) as shown in Equation 9:

$$\lim_{t\to \infty }\left\langle \right\|r_{i}\left(t\right)-r_{i}\left(0\right){\|}^{2}\rangle_{i\in A}=6D_{A}t.$$ (9)

**FIGURE 7:**
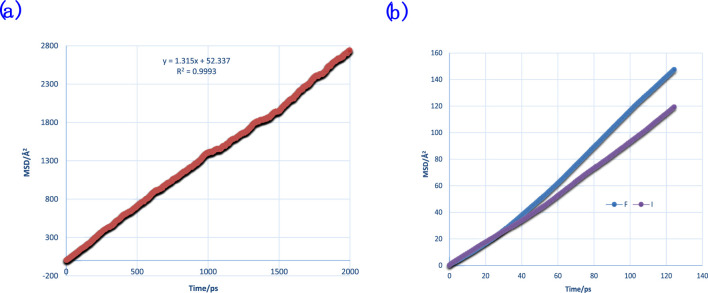
MSDs of water **(A)** and anions **(B)** of F^−^ and I^−^ for comparison with binding energy and DAFs.

In this research, we used the former method to calculate diffusion coefficients. The diffusion coefficient of molecules that build the thin film was examined. The MSDs of water and ions are shown in Figure 7.

The surface tension and surface electrostatic potential of the thin films were also examined and can be referred to in Supplementary Materials.

### 3.2 Film rupture and critical thickness in MD

In real thin film balance experiments, the thin film would sometimes rupture after a period of time, and this time usually can be regarded as the lifetime of the film. Determination of the critical thickness can be based on the average density of the thin film if the film has some holes, as rupture is standard when a film thins. The rupture process could be evaluated using critical rupture time (CRT) or critical thickness, where the critical thickness was related to the ratio of *lateral size*/*thickness* at a certain running time. Generally, when the ratio is high, the rupture would happen more easily.

First, a pure water film was applied to see whether it would rupture after a certain running time. A 3 nm × 3 nm × 1.15 nm pure water film was found to not rupture after 4–5 ns. The pure water thickness was reduced to 1.1 nm, and it was still complete without holes after 4 ns. This suggested that for a lateral size of 3 nm × 3 nm, the critical thickness for pure water film may not be larger than 1.0–1.1 nm. Next, a low concentration of NaCl (9 Na^+^ + 9 Cl^−^) was introduced to the pure water film, and the film with salt still appeared to be intact after 4 ns.

Following a similar methodology as the above procedure, the films of pure water or water with salt were studied, and the final states of the films were collected after 14 ns run time. The films with more than 980 molecules (1,020, 1,085, and 1,250 groups) were all unbroken. Some typical film rupture states (∼1.2 nm thickness) after running for 14 ns are listed in Table 1. Similar to the non-polarizable model, when the films were thick enough, they would not rupture after running for a very long time. Surface tension would also increase with added salts. It was found that at low concentrations, NaF had a breaking effect on the film.

**TABLE 1:** Film (5 nm × 5 nm) rupture states using polarizable ion–water potential with or without salts.

Water film	H_2_O	920	940	950	980	1,020
		B	U	61.3 U	U	U
Films with salts	H_2_O:ions	900:20	920:20	**930:20**	960:20	990:30
	NaF	B	B	**63.9 U**	U	U
	NaCl	B	U	**62.1 U**	U	U
	NaI	B	U	**61.5 U**	U	U

U: unbroken films. B: film broken; for a film with NaCl 960:20, 960:20 means the film consists of 960 H_2_O and 20 ions (10Na^+^, 10Cl^−^). Bold values in the table indicate the critical thickness.

**FIGURE 8:**
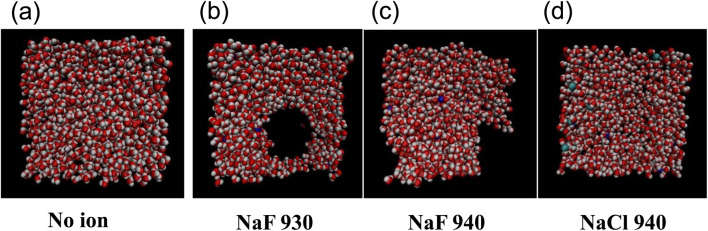
Snapshots for the water 940 film **(A)**, the NaF 930 and NaF 940 films **(B, C)**, and the NaCl 940 film **(D)**.

Figure 8 shows the final states of the pure water film and the films with NaF and NaCl. It showed that at the same lateral size and film thickness, for example, 5 nm × 5 nm with 940 molecules, NaCl exhibited a weaker breaking effect than NaF.

The comparison between a non-polarizable model and a polarizable model was that both models showed that salts in relatively low concentrations destabilized the films. At room temperature in MD, for example, 300 K, the water films with a thickness of 1.5–2 nm did not rupture. This is because in a computer simulation, the finite box size limits the long wavelength fluctuations (Bhatt, D.; Newman, J.; Radke, C. *J Phys Chem B* 2002, 106, 6,529–6,537). However, when the lateral size increased to be big enough, the films would rupture.

### 3.3 Factors contributing to film stability and rupture in MD

#### 3.3.1 Binding energy

**FIGURE 9:**
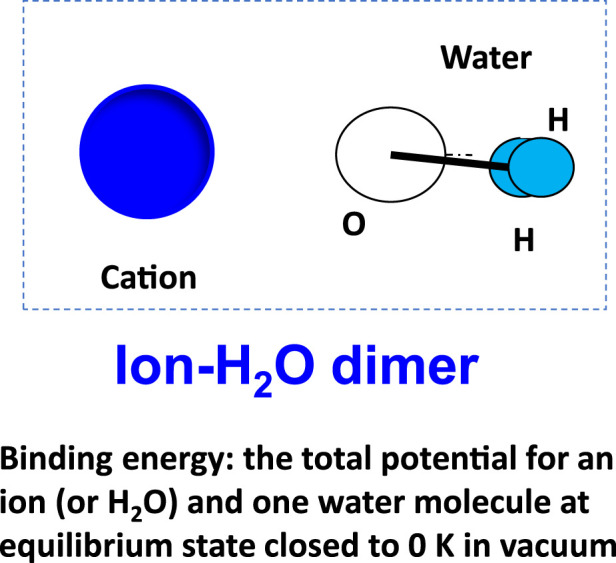
Binding energy of ion–water (or water–water) dimer as insight into the molecular interactions contributing to film rupture and stability.

Binding energy is the total potential energy of a dimer, such as an ion–water (Figure 9), after equilibrium. The binding energy can open the way to a detailed study of the underlying cause for some phenomena at a molecular level. It could also be obtained from *ab initio* and from quantum mechanics (QM) calculations. The binding energies of typical dimers are presented in Table 2.

**TABLE 2:** Binding energy (kcal/mol) between ion and one water molecule for different models.

	Non-polarizable model	Polarizable ion–water potential	QM (Hashimoto and Kamimoto, 1998; Feller et al., 1994; Kim et al., 1995; Lee et al., 1999; Park et al., 2004; Kołaski et al., 2007; Kim et al., 2000; Lee and Kim, 2001; Glendening and Feller, 1995)
F^−^	−18.55	−22.72	−19.8
Cl^−^	−12.63	−14.65	−13.1
Br^−^	−11.67	−12.86	−11.9
I^−^	−10.27	−10.28	−10.2
Na^+^	−24.47	−25.20	−24.3
Water–water	−6.78	−4.80	–

The absolute binding energy of Na^+^-water was stronger than that of halide ions. This also could be validated by the result of RDF for cations and anions. In the comparison of F^−^ and Br^−^, the trend is that F^−^ appeared to be “tighter.” In turn, these quantities lead to differences in molecular distribution profiles.

From Table 2, it was seen that the F^−^–water interaction was relatively larger than that of I^−^–water. In addition, the I^−^–water interaction was a little larger than the water–water interaction. In the MD simulation performed, NaF indeed had a much stronger breaking effect than other ions.

#### 3.3.2 Molecular interaction within thin films by DAF

Similar to binding energy, DAF may be an important quantity that could be applied to study the molecular interaction directly by simulations. The theory of correlation functions is well-established, and the definition of the autocorrelation function (ACF) $C_{f}\left(t\right)$ for a property is shown in Equation 10:

$$C_{f}\left(t\right)={\langle f\left(\xi \right)f\left(\xi +t\right)\rangle }_{\xi },$$ (10)

where the notation on the right-hand side indicates averaging over time origins. It is also possible to compute the cross-correlation function from two properties, as shown in Equation 11:

$$C_{fg}\left(t\right)={\langle f\left(\xi \right)g\left(\xi +t\right)\rangle }_{\xi }.$$ (11)

The integral of the correlation function over time is the correlation time as shown in Equation 12:

$$\tau_{f}=\int_{0}^{\infty }C_{f}\left(t\right)dt.$$ (12)

Here, correlation functions are calculated based on data points with discrete time intervals so that the ACF from an MD simulation is shown by Equation 13:

$$C_{f}\left(j\Delta t\right)=\frac{1}{N-j}\sum_{i=0}^{N-1-j}f\left(i\Delta t)f(\left(i+j\right)\Delta t\right),$$ (13)

where *N* is the number of available time frames for the calculation. There are some important varieties of the ACF, for example, the ACF of a vector **P**, as shown in Equation 14:

$$C_{p}\left(t\right)=\int_{0}^{\infty }P_{n}(\cos∠\left(p\left(\xi \right),p\left(\xi +t\right)\right)d\xi ,$$ (14)

where ***P***_*n*_ is the *n*th-order Legendre polynomial. Such correlation times could be determined experimentally using, for example, NMR or other relaxation experiments. These correlations can be calculated using the first- and second-order Legendre polynomials. This can also be applied to autocorrelation, such as dipole autocorrelation.

The dipole correlation function for particles of type ***A*** is calculated as shown in Equation 15:

$$C_{\mu }\left(\tau \right)=\langle \mu_{i}\left(\tau \right)\cdot \mu_{i}\left(0\right)\rangle_{i\in A},$$ (15)

where $\mu_{i}=\sum_{j\in i}r_{j}q_{j}$ .

**FIGURE 10:**
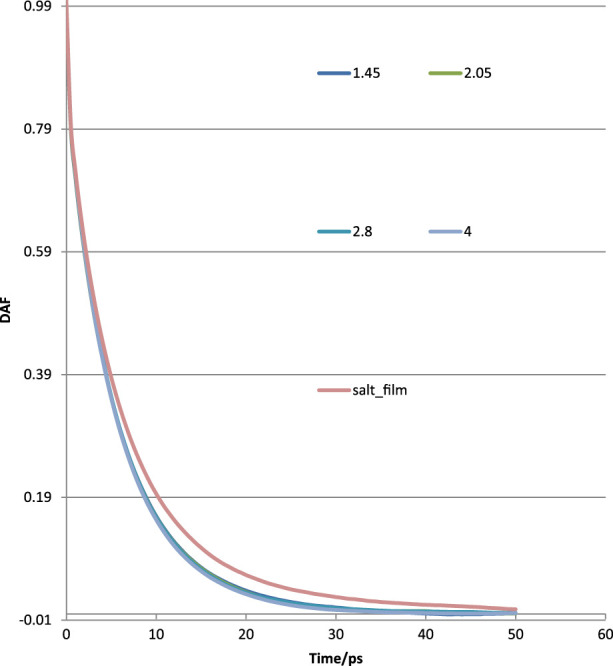
Water–water interactions in terms of DAFs at various thicknesses for thin films without and with ions.

The calculated DAFs at various film thicknesses at the same lateral size of 4 nm × 4 nm for pure water films and with ions are shown in Figure 10. It could be observed that the DAFs of thin film with ions (orange color curve) are significantly different than that of pure water without ions. This could, in part, explain the influence of ions on water rupture as examined using MD. The DAFs for different thicknesses of pure water films showed no remarkable difference; some showed a larger value at the early running time but a smaller value at a later stage.

The DAFs would generally decay with running time. These lines could be fitted using *exp* (*x*), and the final version can be expressed as DAF = *exp*(–((*x*/P1)^P2)), where *x* is running time in MD, and P1, P2 are fitted tau (τ) and beta (β), as in Supplementary Table S2.

**FIGURE 11:**
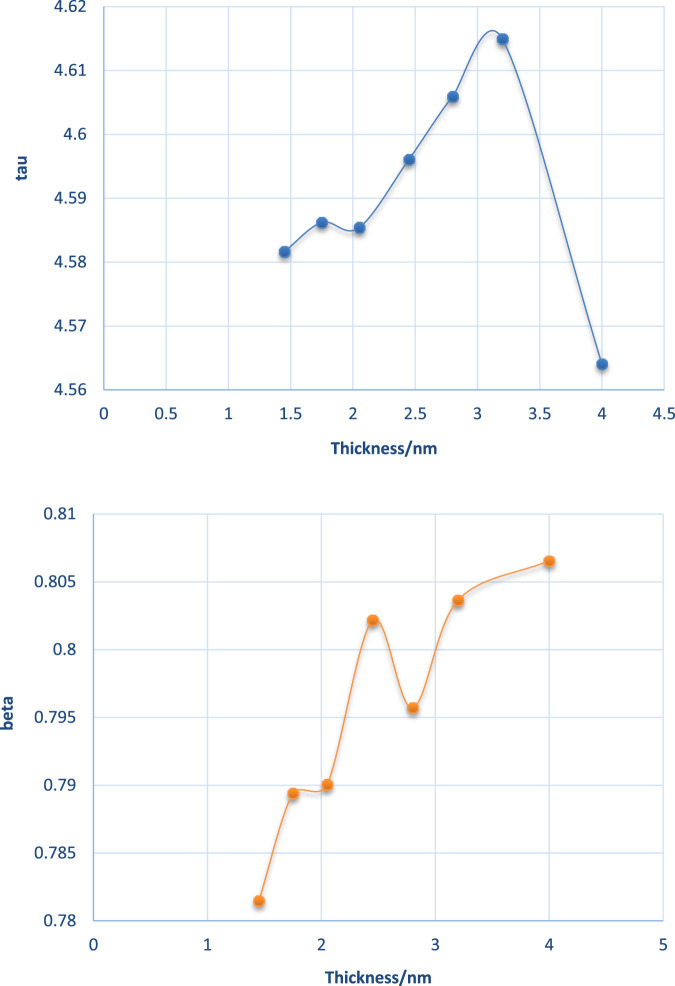
Fitted tau (τ) and beta (β) at various thicknesses for the water–water interaction within thin films.

**FIGURE 12:**
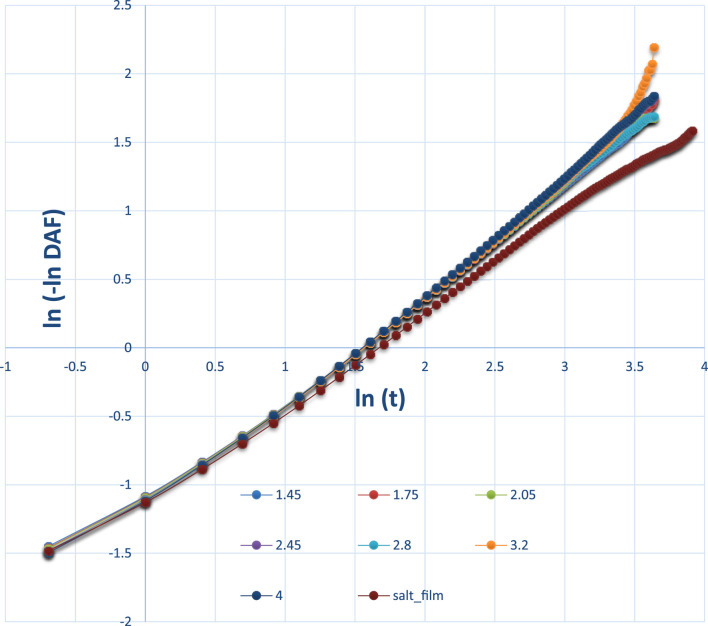
Logarithm style of DAFs at different thicknesses. When the DAF has a negative value, it is not possible to continue to fit the data.

As seen in Figure 10, the DAF curve of the salt film was on the high side of the pure water film. The water–water interaction in salt film may be stronger than that of a pure water film. The fitted tau (τ) and beta (β) for pure water are plotted in Figure 11. The obtained tau (τ) was shown to increase with the film thickness until it reached a film thickness of 4 nm. This may suggest that 4 nm × 4 nm × 4 nm as a cubic size indeed could be regarded as bulk water. Its air–water surface and the interaction of the two surfaces could be negligible as the disjoining pressure is 0 Bar. In order to better study the DAFs at different thicknesses for these water films (at the same lateral size of 4 nm× 4 nm), Figure 10 was transformed to Figure 12 using Logarithm style ln(-ln*DAF*) = A(ln*t*)-B.

Figure 12 also shows the difference between the pure water film and the NaCl film. At a later time, for example, ln*t* at or after 3.5, the orange line for the water film thickness of 3.2 nm was significantly higher than the lines for other thin films. Note that the DAFs at a later stage, for example, 100 ps, will fluctuate and will be close to 0.

**FIGURE 13:**
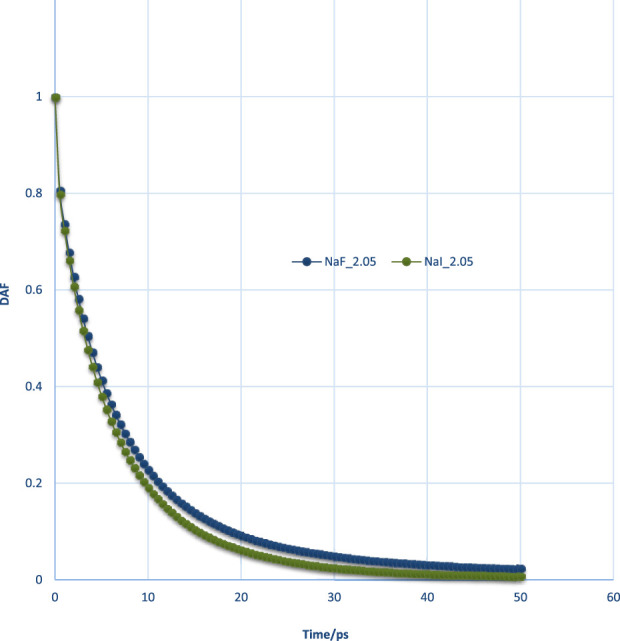
DAFs of thin films for comparison of F^−^ and I^−^ at the same lateral size and thickness conditions.

The effect of different ion types on DAFs was studied and is shown in Figure 13. The DAF within films containing F^−^ was shown to be higher than that of I^−^, exhibiting a stronger water interaction network for a film with F^−^. This, in turn, could be validated with the binding energy data from Table 2. It can also be interpreted by RDFs of the two ion types.

Usually, DAF will monotonically decrease with time. If the DAF value at some time, for example, for a pure water film with a thickness of 3.2 nm after 40 ps, was not monotonically decreasing, or if it was a negative value, the decay time of this film should not be higher than this time point, which indicates that its decay time is shorter than 40 ps. In order to examine the difference between the two different thicknesses, the DAFs must sometimes be compared by subtraction. At the initial stage (<=5 ps), the DAFs will increase with the increase of thickness; however, there was an inflection point after 5 ps.

## 4 Conclusion

Research into the film stability between two bubbles in foams has been motivated by different interests. The behavior of foam films is significant in many areas, such as mineral flotation. As a major mineral enrichment method, flotation is widely used in copper and many major mines worldwide, especially in the separation of fine particles of non-ferrous and rare metals. In this study, the molecular modeling method was applied to study the aqueous thin film characteristics and molecular mechanisms of micro-nano foam stability. The simulation results were extensively analyzed to determine how variation of ion concentration affects interfacial region structure and near-interface properties. The film rupture behavior was investigated, and the factors contributing to thin film stability were also studied in terms of molecular interactions, using qualities such as RDF, binding energy, DAFs, and so on. Salt ions were observed to destabilize the nanofilms at low concentrations. The ability of a salt to break the films depends on the strength of the ion–water interaction and the molecular partition at the film surfaces. This research will advance the state of the art for MD simulation methods, and it will provide a molecular-level understanding of how ions affect bubble merging processes and near-interface stability in liquid water films. The effects of ions on interfacial phenomena in water revealed by this investigation will also contribute to the understanding of biological and environmental processes at a liquid water interface.

## Data Availability

The datasets presented in this study can be found in online repositories. The names of the repository/repositories and accession number(s) can be found in the article/Supplementary Material.
