# Supplementary material for: Surface characteristics and molecular interactions of thin films between bubbles by molecular simulations
Source: Front Chem. 2025 Jan 6;12:1493571. doi: 10.3389/fchem.2024.1493571 (PMC11743664; doi:10.3389/fchem.2024.1493571)
Supplement: Supplementary file 1 [file DataSheet2.docx]

Table S1 Potential parameters for water and ions in the simulation.

| Molecule | Atom type | r_m_/2 (Å) | ε (kcal/mol) | *q* (e) | *α* (Å^3^) |
| --- | --- | --- | --- | --- | --- |
| H_2_O | H | 0 | 0 | 0.519 | 0 |
|  | O | 1.815 | 0.183 | 0 | 0 |
|  | M | 0 | 0 | -1.038 | 1.444 |
| Cs^+^ | Cs | 2.098 | 0.10 | 1 | 2.440 |
| Na^+^ | Na | 1.275 | 0.10 | 1 | 0.24 |
| Cl^-^ | Cl | 2.435 | 0.10 | -1 | 3.690 |
| I^-^ | I | 2.876 | 0.10 | -1 | 6.920 |

Surface tension and surface electrostatic potential

According to classical DLVO theory, the film is regarded as being unstable in the range of thickness for which the disjoining pressure has a positive slope, since film thinning should proceed spontaneously until film rupture occurs. Meanwhile, the disjoining pressure is significantly influenced by the surface tension of thin films at varied thickness.

As analysis above, surface tension of the bulk liquid and surface tension of thin films are significant to the stability of liquid film. It should be noted that the surface tension of the very thin film can be significantly different from that of bulk liquid surface tension. From experiment, it is possible to measure the bulk liquid surface tension of common liquids, referred as *σ*_∞_, it is a constant at a certain condition such as concentration or temperature. But it is unable to measure surface tension of thin films by experimental measurement. From molecular simulation, it is possible to calculate the surface tension of bulk liquids, and also possible to calculate the surface tension of thin films with smaller thickness.

Fig. S3 Procedures for determination of surface tension by molecular simulations.

For the determination of surface tension of liquid, the initial bulk structure with smaller density, with *L*_x_=*L*_y_, was firstly built within NPT ensemble (isothermal-isobaric), after run in NPT for ~1 ns, the correct density corresponding to the liquid was obtained. After equilibrium at NPT, NVT (canonical ensemble) was adopted to relax the system, and then two vapor boxes were added to simulate the vapour-liquid surface as shown in S3. Surface tension was determined by the conventional pressure tensor way [[1](#_ENREF_1)] using molecular simulations, i.e., given by

$\sigma_{\text{f}}=\frac{1}{2}\int_{0}^{L_{z}} \left(P_{N}-P_{T}\right)dt$ (10)

Where, *P_N_* and *P_T_* are pressure tensors, at normal and tangential direction, respectively.

The surface tensions of thin liquid film with ions were obtained by the similar pressure tension method. Surface tension was sensitive, and it is beneficial to capture the trend. The surface tension for liquid films with NaI at different film thicknesses were calculated, the results ranged from 61.9 to 62.4 mN/m. For comparison, the corresponding surface tension at the same condition for liquid films with that of NaCl were also calculated and obtained, the results lie from 63.1 to 63.7 mN/m. At the same condition, the surface tension of liquid films with NaCl was observed to be higher than that of films with NaI.

The electric potential difference across the interface ∆Φ is defined as:

 (11)

The surface potential at the vapor−liquid interface using explicit MD simulations for thin films are also been calculated, and the obtained data had value around -0.49 V for water films. By adding ions, the absolute value decrease slightly, and the value became -0.46~-0.47 V.

Table S2 Fitted tau (τ) and beta (β) at different thicknesses from 1.45 nm to 4 nm with lateral size 4×4 nm.

| Thickness (nm) | 4×4×1.45 | 4×4×2.05 | 4×4×2.8 | 4×4×4 |
| --- | --- | --- | --- | --- |
| τ | 4.58 | 4.59 | 4.61 | 4.56 |
| β | 0.78 | 0.79 | 0.8 | 0.81 |

1. Brown, D. and S. Neyertz, *A general pressure tensor calculation for molecular dynamics simulations.* Molecular Physics, 1995. **84**(3): p. 577-595.
