# Supplementary material for: Surface characteristics and molecular interactions of thin films between bubbles by molecular simulations
Source: Front Chem. 2025 Jan 6;12:1493571. doi: 10.3389/fchem.2024.1493571 (PMC11743664; doi:10.3389/fchem.2024.1493571)
Supplement: Supplementary file 2 [file DataSheet1.docx]

Fig. S1 Typical illustration of foam column structure, (a) separation of polyhedral bubbles by liquid film, (b) foam drainage transform from Kugelschaum to Polydermschaum.

Fig. S2 Methodology for modelling surface interaction of liquid film between bubbles.
